# An interactive mindmap of blood film images for automated malaria diagnosis: a comprehensive metadata repository of available datasets

**DOI:** 10.1093/bioadv/vbaf276

**Published:** 2025-11-04

**Authors:** Itunuoluwa Isewon, Faith Adegoke, Charles B Delahunt, Jelili Oyelade

**Affiliations:** Department of Computer and Information Sciences, Covenant University, P.M.B 1023, Ota, Ogun State, Nigeria; Covenant Applied Informatics and Communication African Centre of Excellence (CApIC-ACE), Covenant University, P.M.B 1023, Ota, Ogun State, Nigeria; Covenant University Bioinformatics Research (CUBRe), Covenant University, P.M.B 1023, Ota, Ogun State, Nigeria; Department of Computer and Information Sciences, Covenant University, P.M.B 1023, Ota, Ogun State, Nigeria; Covenant Applied Informatics and Communication African Centre of Excellence (CApIC-ACE), Covenant University, P.M.B 1023, Ota, Ogun State, Nigeria; Covenant University Bioinformatics Research (CUBRe), Covenant University, P.M.B 1023, Ota, Ogun State, Nigeria; Global Health Labs, Bellevue, WA, United States; Department of Computer and Information Sciences, Covenant University, P.M.B 1023, Ota, Ogun State, Nigeria; Covenant Applied Informatics and Communication African Centre of Excellence (CApIC-ACE), Covenant University, P.M.B 1023, Ota, Ogun State, Nigeria; Covenant University Bioinformatics Research (CUBRe), Covenant University, P.M.B 1023, Ota, Ogun State, Nigeria

## Abstract

**Summary:**

Malaria remains a significant public health challenge, necessitating innovative diagnostic solutions to combat high mortality rates. Here, we conducted a systematic review of malaria microscopy datasets published between 2014 and 2024 by querying Scopus and PubMed databases. We have a curated inventory of 71 malaria blood film image datasets. To further enhance usability, we developed an interactive mind map that visually represents the datasets based on five key features. The **Access** category differentiates between open and controlled access datasets. The **Smear Type** category classifies datasets by blood smear type. The **Stain Type** category organizes datasets by the staining methods used. The **Species Type** highlights the *Plasmodium* species present in the images, while the **Demography** category identifies the geographic origin of datasets. Each dataset has a dedicated page with a detailed description, a citation of the dataset’s publication, and links to databases where it can be downloaded. This mind map has been carefully curated with up-to-date information and will be regularly updated as new datasets become available, ensuring its continued relevance and accuracy.

The mind map is a freely accessible resource, available at https://itunuisewon.github.io/Malaria_Blood_Film_Images/, which enables researchers to identify and utilize diverse datasets for training and validating machine learning algorithms.

**Availability and implementation:**

Not applicable.

## 1 Introduction

Computer-aided diagnosis (CAD) uses computers and computational tools to analyse and interpret medical data for disease diagnosis. Recent studies have highlighted the efficacy of CAD techniques in enhancing diagnostic accuracy. Such studies involve interdisciplinary collaboration among expert pathologists, computer scientists, and mathematicians (Molina *et al.* 2021). Furthermore, these methods have also significantly reduced the manual inspection workload for healthcare professionals by automating various aspects of data analysis.

Malaria remains a public health concern globally (Olasehinde *et al.* 2019). It is caused by the *Plasmodium* parasite, which is transmitted to humans through the bite of female *Anopheles* mosquitoes. Among the species that infect humans, five variants are identified: *Plasmodium falciparum*, *Plasmodium vivax*, *Plasmodium ovale*, *Plasmodium malariae*, and *Plasmodium knowlesi* (Oyewola *et al.* 2022). There has been a concerning increase in malaria cases across 85 nations due to the COVID-19 pandemic, which disrupted healthcare systems (Benjamin *et al.* 2023). The 2024 World Health Organization (WHO) malaria report records 263 million cases and 597 000 deaths in 2023, with Africa bearing the majority of the impact. Children under the age of five are among the groups that are at high risk of malaria infection (WHO 2024).

Early detection is vital for malaria diagnosis to ensure timely and appropriate treatment. However, diagnosing malaria can be particularly challenging in non-endemic regions, where clinicians may not initially consider it as a potential diagnosis due to limited familiarity with the disease, leading to misdiagnosis and inadequate treatment. Accurate detection of malarial parasites in blood samples requires expertise and experience, which laboratory technicians in these regions may lack, increasing the likelihood of diagnostic errors (Rehman *et al.* 2020). In endemic regions, healthcare providers face a heavy workload, which can compromise their diagnostic efficiency and accuracy (Rees-Channer *et al.* 2023). Malaria diagnosis typically includes methods such as microscopy, rapid diagnostic tests (RDTs), clinical evaluations, and molecular techniques like polymerase chain reaction (PCR) (Ojurongbe *et al.* 2013). However, each of these methods has its drawbacks and limitations: PCR requires costly equipment and infrastructure, while RDTs have high detection thresholds and may fail to identify certain *Plasmodium* strains with HRP2 deletions (Prosser *et al.* 2021, Alnussairi and İbrahim 2022).

Globally, microscopy is a well-established technique for malaria diagnosis (Aqeel *et al.* 2023). Despite its reliability, this method is time-consuming and labour-intensive, and the number of skilled professionals using it is insufficient to address the demands imposed by the disease, particularly in resource-constrained settings. Employing computer-aided diagnostic tools can accelerate the process and provide parasitologists with an opportunity for a second opinion. One key advantage of automated malaria microscopy is in quantifying parasitaemia (parasite density), enabling the calculation of clearance curves that help monitor drug-resistant strains of the *Plasmodium* parasite (Delahunt *et al.* 2024). While numerous algorithms have been proposed for detecting malaria in Giemsa-stained blood films, only a small fraction have undergone field validation, and none to date have been integrated into clinical practice.

This paper provides a comprehensive catalogue of publicly available malaria image datasets to aid researchers in building reliable and effective automated malaria diagnosis models. The following sections first detail the microscopy method for malaria diagnosis and then present a compilation of malaria blood film datasets derived from a systematic review of existing literature.

## 2 Malaria microscopy

The process of malaria microscopy involves examining stained thick and thin blood films under a microscope to detect and identify malaria parasites. The sensitivity of this technique is reduced in cases of low parasitemia, emphasizing the necessity for skilled personnel to handle the staining process properly. Furthermore, microscopy demands considerable time and labour.

### 2.1 Blood sample collection

Blood samples are typically collected via a finger prick (or venipuncture in specific circumstances). A sterile lancet is used to puncture the third finger of the patient’s hand, following alcohol swabbing to clean the site. A drop of blood is then transferred to a slide to prepare thick and thin films. In cases requiring venipuncture, a larger sample of venous blood is drawn and stored in tubes containing anticoagulants, subsequently used for preparing the blood films (WHO 2016a).

### 2.2 Film preparation

Blood samples from the patients are used to prepare thick and thin blood films for malaria diagnosis using microscopy. In a thin film, red blood cells are arranged in a single layer, allowing for the observation of the parasite within the red blood cells (RBCs). This film is fixed with methanol and is useful for identifying the species of malaria parasite, determining life cycle stages, and quantifying high levels of parasitemia. However, its sensitivity decreases at low parasite densities. The distinction between RBCs and other blood components is clearer due to improved visibility (Rehman *et al.* 2018). To prepare the thin film, a “spreader” slide is used to spread the blood drop quickly and smoothly, creating a feathery edge.

A thick blood film is more sensitive in detecting parasites than a thin blood film. It is prepared by swirling the blood drop, resulting in a denser film that allows for a larger volume of blood to be examined. In this film, RBCs are ruptured, leaving only the white blood cells (WBCs), platelets, and parasites visible. One challenge with this method is distinguishing the parasites from other blood components, i.e. a higher frequency of artefacts (Maturana *et al.* 2022). Unlike the thin film, it is not fixed in methanol and can also be used to quantify parasitemia (Musumeci 2014). Figure 1 shows a pictorial representation of the thick and thin Giemsa-stained blood films.

**Figure 1.**
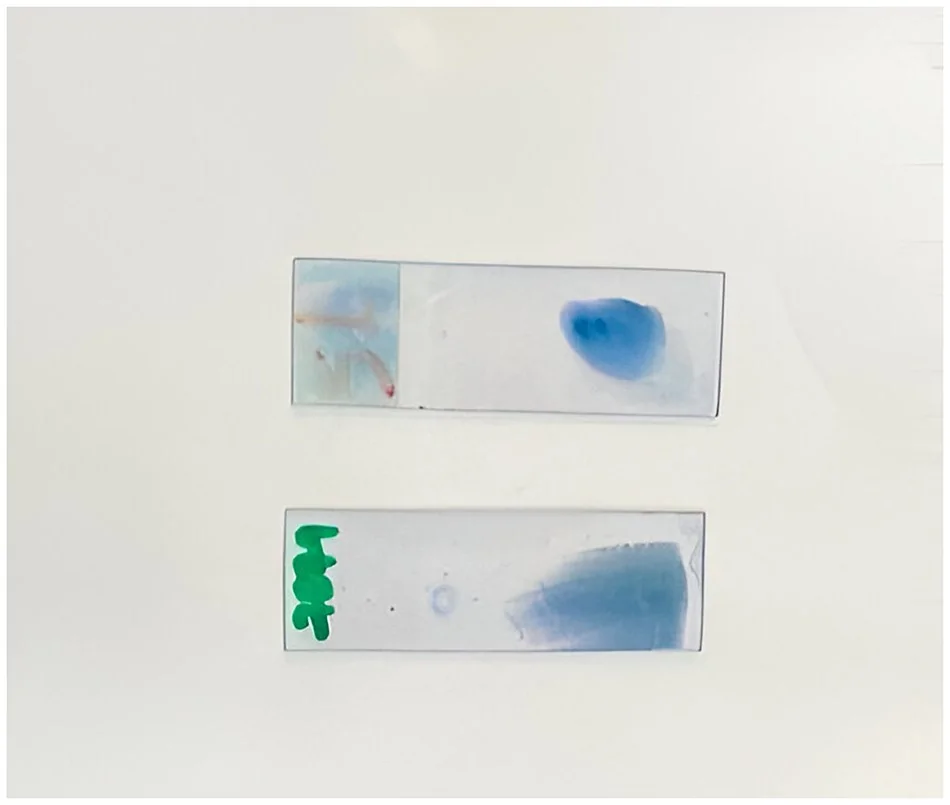
Giemsa-stained blood film.

### 2.3 Staining of slides

In the microscopy method for malaria diagnosis, histological staining is a crucial step in preparing tissue samples. Enhancing their contrast, makes them more visible and easier to examine under the microscope. The process of histological staining consists of five key stages: fixation, processing, embedding, sectioning, and staining (Horobin 2011). The Romanowsky stain, widely used since its introduction in the 1890s, is one of the primary stains used in this process (Musumeci 2014). Methylene blue binds to acidic molecules, while eosin binds to neutral or basic cellular compounds. As a result, the nuclear material (DNA) stains purple, while the cytoplasm stains blue. As red blood cells have no nucleus, while examining a clean blood film, only white blood cells and *Plasmodium* parasites contain nuclei, thus stain purple. The cytoplasm of *Plasmodium* parasites typically appears distinct and stains blue. Variants of the Romanowsky stain include Giemsa, Leishman, Field, May Grunwald-Giemsa (MGG), Wright, and Jaswant Singh-Bhattacharji stains, each with its unique modifications. The Giemsa stain is widely favoured for its stability and consistent quality across varying temperatures, while Leishman’s stain is also commonly used in malaria diagnosis. The Field stain is user-friendly, has a short staining time, and is cost-effective. However, if used by inexperienced personnel, there is a higher risk of false positives, as improper staining can result in an increased number of artefacts (Janiesch *et al.* 2021). Even with correct application, the resulting blood film can vary in appearance due to factors such as the stain’s age, concentration, and other variables. The Jaswant Singh-Bhattacharji (JSB) stain, developed in India, offers a quick and inexpensive solution. However, its tendency to fade over time makes it unsuitable for archived slides (Kalinin *et al.* 2024).

### 2.4 Imaging

Malaria parasites are minuscule (with morphological details around 0.1 µm), so a high-resolution system is required for accurate diagnosis. Stained slides are typically examined using an optical (light) microscope with oil immersion at 100× magnification for high-resolution imaging. Alternatively, a microscope with a 40× objective (NA = 0.75) may be used for lower-magnification overviews or when oil immersion is not required (Horning *et al.* 2021). The image is then adjusted for optimal clarity. Microscopy images of malaria-infected thick and thin blood films are shown in Figs 2 and 3, respectively. The thick blood film can be used to detect parasite density, while the thin blood film shows the parasite morphology in detail and can be used to identify different species of the parasite.

**Figure 2.**
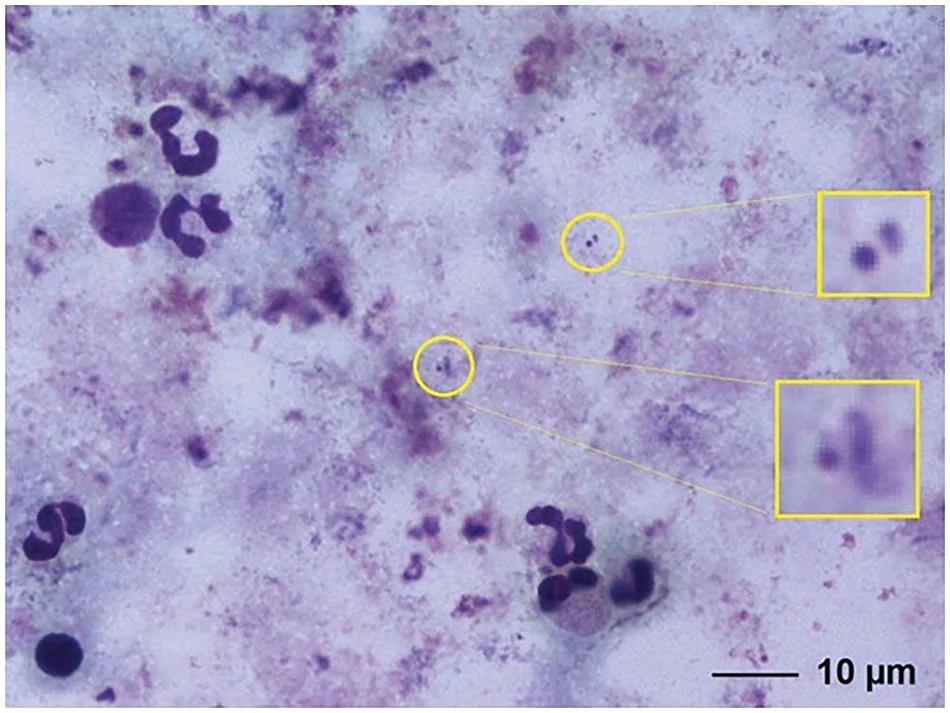
Thick Giemsa-stained blood film. Permission from Mehanian et al., Computer-automated malaria diagnosis and quantitation using convolutional neural networks, ICCV 2017 (Mehanian *et al.* 2017).

**Figure 3.**
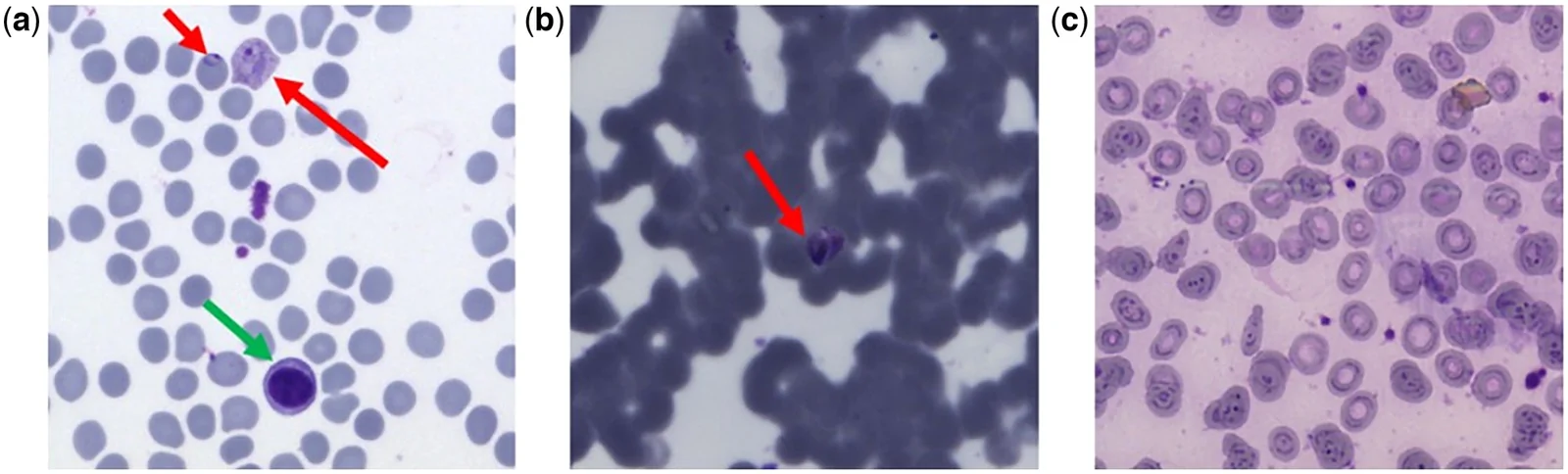
Thin blood film field of views. (a) an ideal field of view. (b) clumped red blood cells. The green arrow shows a white blood cell, and the red arrows show red blood cells infected by the *Plasmodium* parasites. Other purple-stained objects are artefacts. Permission from Delahunt *et al.*, Full-automated patient-level malaria assessment on field-prepared thin blood film microscopy images, IEEE GHTC 2019 (Delahunt *et al.* 2019).

### 2.5 Malaria diagnosis

As outlined in the WHO malaria microscopy standard operating procedure (WHO 2016b,c), a drop of immersion oil should be applied to the thick blood film, with the 100× objective lens making contact with the oil. The fine adjustment knob is then used to focus the slide, which should be examined systematically. For a slide to be considered non-parasitized, at least 100 fields of view must be examined without the detection of a parasite. If parasites are found, they should be counted along with WBCs until 200 WBCs have been counted. The formula in (1) below is used to calculate parasitemia:


(1) $$\frac{\mathrm{Plasmodium}\;\mathrm{parasite}\;\mathrm{counted}\;\times 8000}{\mathrm{WBC}\;\mathrm{counted}}=\mathrm{parasites}/\mu L\;\mathrm{blood}$$

The thin blood film should be examined at its feathery edge to identify parasite species after reviewing the thick blood film.

## 3 Machine learning for malaria diagnosis

Machine learning algorithms and computer vision techniques have been leveraged to aid malaria diagnosis. Machine learning enables a computer program to optimise tasks and improve performance metrics through experience and iterative learning (Rabbani *et al.* 2013). It has been extensively used in medical imaging, employing traditional methods such as image acquisition, pre-processing, segmentation, feature extraction, feature selection, and classification (Molina *et al.* 2021). In the past, researchers have relied on hand-crafted features like histograms of gradients, Haralick’s texture features, RGB values, co-occurrence matrices, shape descriptors such as area, perimeter, compactness ratio, eccentricity, and bending energy, along with local binary patterns (Poostchi *et al.* 2018).

Additional methods include Support Vector Machine (SVM) (Linder *et al.* 2014), Decision Tree (Rabbani *et al.* 2013), Naive Bayes (Das *et al.* 2015), Linear Discriminant (Park *et al.* 2016), and K-Nearest Neighbour (KNN) (Malhotra *et al.* 2017). Shallow machine learning models have also been used in image classification for malaria diagnosis. With advancements in deep learning (DL), artificial intelligence has significantly improved in overall capabilities and performance. Deep learning has gained popularity and has shown results in domains such as Natural Language Processing and Computer Vision (Haralampieva *et al.* 2020). This is due to its automated feature extraction within vast data frameworks, enabling DL models to learn autonomously, much like the human brain.

Among the various deep learning methods used for automatic diagnosis, the Convolutional Neural Network (CNN) is particularly prevalent. CNN excels in image classification due to its speed and efficiency. It outperforms other methods by adopting a hierarchical learning structure that can be robustly trained once the model structure is matched to the input features. Compared to traditional classifiers, CNN is a flexible learning framework that does not require prior feature extraction. The CNN architecture is constructed with the convolutional, pooling, and fully connected (FC) layers (Pan *et al.* 2018). The input images are first processed through the convolutional layer, where convolution with a specific *N* × *N* filter extracts relevant image features. The pooling layer, usually positioned between two convolutional layers, reduces the image dimensions. Finally, the FC layer connects neurons based on the sum of the previous features to generate a specific output, often serving as a classifier (Hcini *et al.* 2022). CNN architectures commonly used are VGGNet (Simonyan and Zisserman, 2015), GoogleNet (Szegedy *et al.* 2014), ResNet (He *et al.* 2015), and AlexNet (Krizhevsky *et al.* 2012).

The ring stage of *Plasmodium falciparum* is a primary target in drug resistance research because it is during this stage that the parasite is most vulnerable to key antimalarial drugs. Understanding the mechanisms of drug resistance at this stage, such as mutations in the Pfkelch13 gene associated with artemisinin resistance, is critical for developing effective treatment strategies (Mehanian *et al.* 2017, Delahunt *et al.* 2019) developed a framework using the ring stages of *Plasmodium* for parasite quantitation. They also categorized all late stages together for species identification. For optimal processing and precise object detection, a combination of techniques was used in a series, beginning with an initial detector. This distractor filter uses basic, manually designed features in conjunction with a CNN. The resulting output is a combination of patient-level quantitation and species identification predictions. Manescu *et al.* (2020) proposed a classifier called the Deep Malaria Convolutional Neural Network (DeepMCNN), which aggregates the number of *Plasmodium* parasites and white blood cells to estimate parasitaemia. With a positive predictive value (PPV) of 92% and a negative predictive value (NPV) of 90%, the system showed improved accuracy in detecting low parasitaemia levels, proving its effectiveness in adult malaria diagnosis.

In the research report by Kassim *et al.* (2021), Plasmodium VF-Net was proposed for malaria diagnosis on both patient and image levels using thick blood smears. It first determines the presence of infection and then identifies whether the parasite is *P. falciparum* or *P. vivax*. The study employed a Mask R-CNN (Regional-Convolutional Neural Network) to detect parasites and a ResNet50 classifier to minimise false positives. In the study conducted by Yu *et al.* (2023), the NLM Malaria Screener, an app developed for malaria diagnosis, was evaluated. The research was carried out in Sudan with 190 patients, and the Malaria Screener app was used to analyse Giemsa-stained blood smears, while expert microscopy and nested PCR were used as reference standards for comparison. The results indicated that the application achieved a 74.1% accuracy rate for detecting *P. falciparum*, meeting the WHO Level 3 criteria for parasite detection. Additionally, a novel algorithm named Plasmodium VF-Net achieved an 83.1% accuracy when compared to expert microscopy, and 81.0% accuracy when matched against PCR, meeting the WHO Level 2 criteria for detecting both *P. falciparum* and *P. vivax*. These findings suggest that both the Malaria Screener and Plasmodium VF-Net show promise as tools for malaria diagnosis, though further improvements are necessary to enhance their accuracy, identify malaria species, and count the number of parasites. For further insights into critical aspects of developing machine-learning models for malaria diagnosis, please see Delahunt *et al.* (2024).

Data plays a vital role in the development and performance of automated diagnostic systems for malaria. While machine learning models, especially deep learning frameworks, have shown promise in detecting and quantifying parasitemia, their accuracy and generalizability are heavily dependent on the quality and diversity of training data. A larger, well-annotated, and geographically diverse collection of microscopy image datasets is essential to improve model robustness.

## 4 Available blood film images

Seeing the key factor data plays in developing this automated diagnostic system, a systematic review was conducted to look through publications in this domain using a defined search strategy. The review methodology adhered to the guidelines outlined in the Preferred Reporting Items for Systematic Review and Meta-Analysis (PRISMA) protocol (Page *et al.* 2021).

### 4.1 Eligibility criteria

The eligibility criteria for the study, which determine the publications to be included, are shown in Table 1. Each study from the structured search was assessed for image dataset inclusion.

**Table 1.** The eligibility criteria for the study.

Inclusion criteria	Criteria
IC1	The study was published between 2014 and 2024.
IC2	The study involves the automated diagnosis of Malaria targeting microscopy.
IC3	The study is published in a conference, journal, or book chapter.
IC4	The full text of the study is available.
IC5	The study is in the English Language.

Exclusion criteria	Criteria
EC1	The study did not give sufficient information about the dataset.
EC2	The study is not focused on automated microscopy.

### 4.2 Search strategy

To ensure comprehensive data collection, a search strategy was employed to craft precise search terms and pinpoint important databases for collecting relevant documents. Using a query string tailored to the study’s objectives, articles were retrieved from Scopus and PubMed, as these databases host peer-reviewed content and other pertinent information. The search query included terms like “automated malaria diagnosis” and “malaria image dataset,” with MeSH terms applied for PubMed searches. As illustrated in Fig. 4, the search for datasets followed the PRISM guidelines, and the figure shows the search results and processing steps from 2014 to 2024. An overview of publications over the years of focus is shown in Fig. 5.

**Figure 4.**
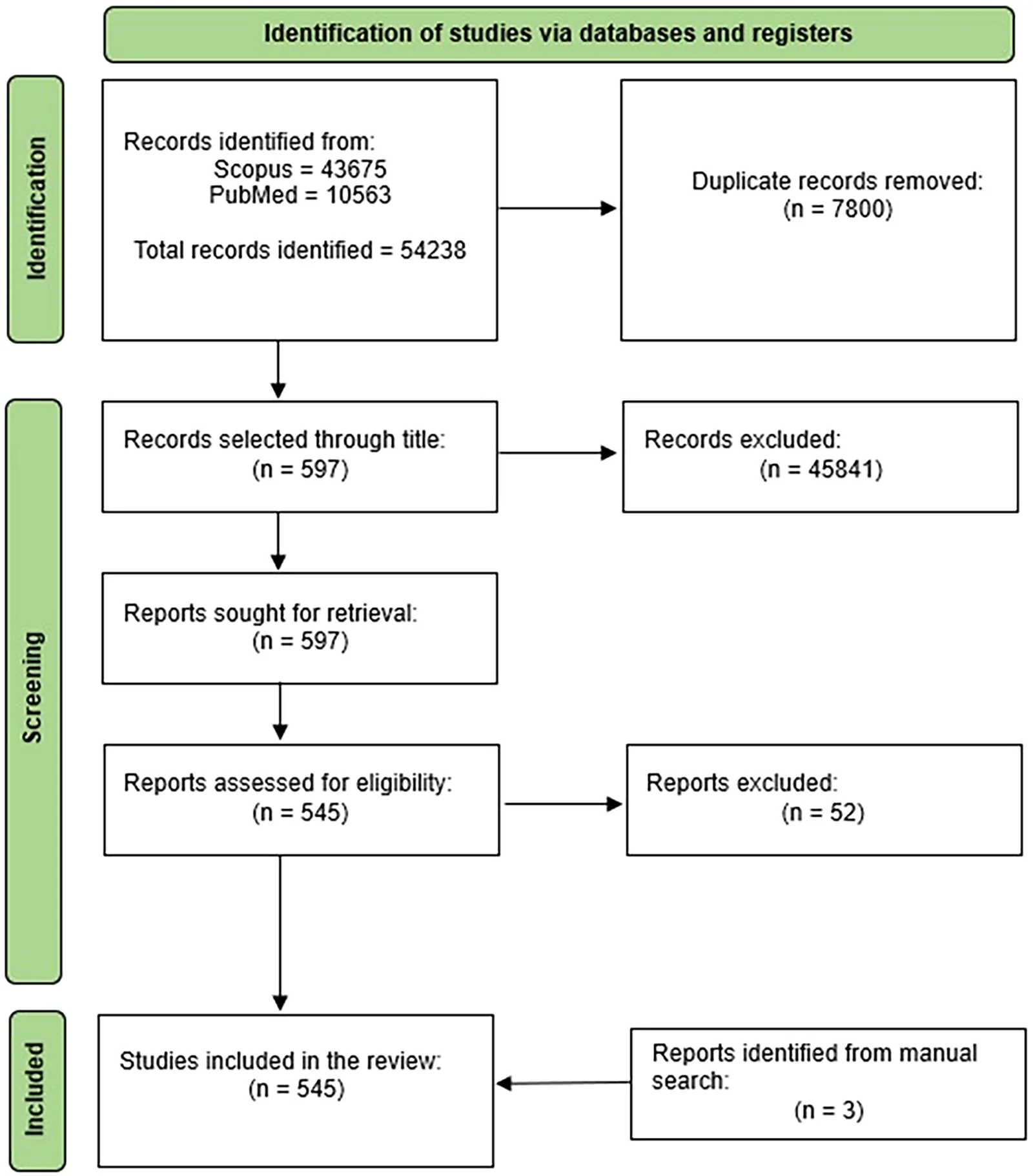
PRISMA flowchart showing the screening process.

**Figure 5.**
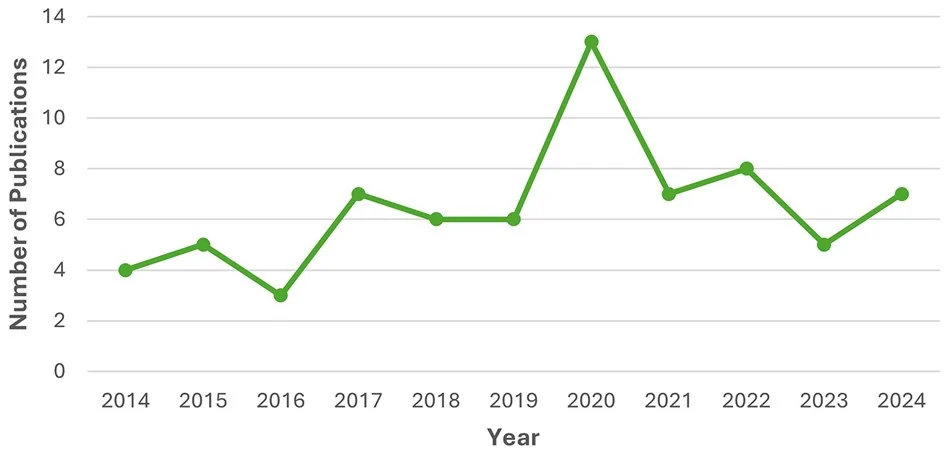
Summary of all publications included in the study by year of publication.

### 4.3 Summary of identified datasets

From the systematic review, 54 238 records were retrieved from searches conducted on PubMed and Scopus databases. After merging the records, 7800 duplicates were identified and removed. Subsequently, 45 841 records were screened out based on their titles, narrowing down the list to 597 articles for further review. The screening process, based on the eligibility criteria provided in Table 1, resulted in 545 studies being included. An additional three records were added through a manual search. The study identified 71 blood film image datasets, summarized in Table 2.

**Table 2.** Summary of available malaria image datasets.^a^

Identifier	Country	*Plasmodium* species	Film type	Optical Train	Stain type	No of Patients	No of images	Type of Image	Publicly Available?
D1 (Yu *et al.* 2023)	Sudan	Pv, Pf	Thick and Thin	Microscope + smartphone	Giemsa	95	Thick—2944 Thin—875	FoVs	Yes
D2 (Quinn *et al.* 2014)	Uganda	Pf	Thick	Microscope + Camera	Field	133	2703	FoVs	Yes
D3 (Fong Amaris *et al.* 2022)	Columbia	Pv	Thick	Microscope	Modified Romanowsky	42	420	FoVs	Yes
D4 (de Souza Oliveira *et al.* 2022)	Brazil	Pv	Thick	Microscope	Giemsa	NR	676	FoVs	AOR
D5 (Arshad *et al.* 2022)	Pakistan	Pv	Thin	Microscope	Giemsa	NR	345	FoVs	AOR
D6 (Nakasi *et al.* 2021)	Uganda	Pf	Thick	Microscope + smartphone	Field	NR	903	FoVs	Yes
D7 (Manescu *et al.* 2020)	Nigeria	Pf	Thick	Microscope	Giemsa	299	NR	FoVs	Yes
D8 (Delgado-Ortet *et al.* 2020)	Spain	Pf	Thin	Microscope + Camera	May-Grunwald-Giemsa	NR	517	FoVs	Yes
D9 (Loddo *et al.* 2019)	Switzerland	Pf, Pm, Po, Pv	Thin	Microscope	Giemsa	NR	Pf—122 Po—29 Pm—37 Pv—46	FoVs	Yes
D10 (Rajaraman *et al.* 2018), (Yang *et al.* 2020)	Bangladesh	Pf	Thick and Thin	Microscope + smartphone	Giemsa	200	Thick—1883 Thin—965	Cells images and FoVs	Yes
D11 (Kassim *et al.* 2021)	Bangladesh	Pv	Thick	Microscope + smartphone	Giemsa	200	NR	FoVs	Yes
D12 (Kanafiah *et al.* 2022)	Malaysia	Pk, Pf, Pv	Thin	Microscope + Camera	NR	NR	300	FoVs	AOR
D13 (Kuo *et al.* 2020)	Taiwan	Pf	Thin	Microscope	Giemsa	36	8145	FoVs	Yes
D14 (Aris *et al.* 2021)	Malaysia	Pf, Pv	Thick and Thin	Microscope	NR	NR	Thick—200, Thin—400	FoVs	No
D15 (Maity *et al.* 2020)	India	Pf, Pv	Thin	Microscope	NR	NR	210	FoVs	Yes
D16 (Aladago *et al.* 2019)	Ghana	Pf	Thin	Microscope	Giemsa	NR	403	FoVs	No
D17 (Dave, 2018)	India	Pv	Thick	Microscope + Camera	Giemsa	5	87	FoVs	No
D18 (Delas Peñas *et al.* 2018)	Philippines	Pv, Pf	Thin	Microscope + Camera	NR	NR	Pv = 142 Pf = 221	FoVs	No
D19 (Dantas Oliveira *et al.* 2018)	Spain	Pf	Thin	Microscope + Camera	Giemsa	NR	50	FoVs	No
D20 (Devi *et al.* 2018)	India	Pv, Pf	Thin	Microscope	Leishman	400	1302	FoVs	No
D21 (Rosado *et al.* 2017)	Portugal	Pf, Po, Pm	Thin	mSmartScope prototype + Smartphone	Giemsa	7	566	FoVs	No
D22 (Rosnelly *et al.* 2017)	Indonesia	Pf, Pm, Pv	Thin	Microscope	Giemsa	NR	600	FoVs	No
D23 (Khan *et al.* 2017)	NR	Pv	Thick and Thin	Microscope	Leishman	118	330	FoVs	No
D24 (Preedanan *et al.* 2016)	Thailand	Pf	Thin	Microscope + Camera	Giemsa	NR	15	FoVs	No
D25 (Cruz *et al.* 2016)	Philippines	Pf, Pv	Thin	Microscope + Camera	Giemsa	21	267	FoVs	No
D26 (Abdul-Nasir *et al.* 2015)	Malaysia	Pv	Thin	Microscope + Camera	Giemsa	NR	125	FoVs	No
D27 (Das *et al.* 2015)	India	Pf, Pv	Thin	Microscope	Leishman	NR	750	FoVs	No
D28 (Molina *et al.* 2020)	NR	NR	Thin	Microscope + Camera	Giemsa	87	NR	FoVs	No
D29 (Fu *et al.* 2023)	China	Pf, Pv, Pm, Po	Thin	NR	NR	100	NR	Blood slice images	No
D30 (Linder *et al.* 2014)	Finland	Pf	Thin	Microscope + Camera	Giemsa	47	42 787	FoVs	Yes
D31 (Nugroho *et al.* 2014)	Indonesia	Po	Thin	NR	Giemsa	NR	NR	FoVs	No
D32 (Liu *et al.* 2023)	Sierra Leone	NR	Thin	Microscope + smartphone	Giemsa	NR	177	FoVs	Yes
D33 (Loh *et al.* 2021)	NR	Pf 3D7 strain	Thin	Microscope + Camera	Giemsa	NR	297	FoVs	No
D34 (Acherar *et al.* 2022)	France	Pf	Thin	Microscope + smartphone	Giemsa	202	1250	FoVs	No
D35 (Salamah *et al.* 2019)	Indonesia	NR	Thick	Microscope	NR	NR	30	fov	No
D36 (Sengar *et al.* 2022)	Pakistan	NR	Thin	Microscope	Giemsa	NR	345	Cell images	No
D37 (Yoon *et al.* 2021)	Korea	Pf, Pv	Thick and Thin	Microscope	Giemsa	71	NR	FoVs	No
D38 (Davidson *et al.* 2021)	NR	Pf 3D7, NF54, DD2, and D10	Thin	Different Equipment	Giemsa	NR	108	FoVs	No
D39 (Sunarko *et al.* 2017)	NR	NR	Thin	Microscope	Giemsa	7	NR	FoVs	No
D40 (Zhong *et al.* 2023)	Sudan	Pf, Pv	Thick	Microscope + smartphone	Giemsa	NR	111	FoVs	No
D41 (Wang *et al.* 2023)	China	Pf, Pv, Pm, Po, Pk, Pc	Thin	Microscope + camera	Wright-Giemsa	380	Pf—4405 Pv—3030 Pm—225 Po—117 Pk—4630 Pc—139	FoVs	No
D42 (Hartati *et al.* 2019)	Indonesia	Pm, Pv, Pf	Thin	NR	Giemsa	NR	600	FoVs	No
D43 (Nugroho *et al.* 2021)	Thailand	Pf	Thin	Microscope + camera	Giemsa	NR	280	FoVs	No
D44 (Sampathila *et al.* 2018)	India	Pv	Thin	Microscope + camera	Leishman	NR	143	FoVs	No
D45 (Nugroho *et al.* 2019)	Indonesia	Pv	Thin	NR	NR	NR	73	FoVs	No
D46 (Kudisthalert *et al.* 2020)	Thailand	Pf	Thin	Microscope + camera	Giemsa	NR	23 248	Cell images	No
D47 (Shi *et al.* 2020)	China		NR	NR	NR	NR	217	Cell images	No
D48 (Vijayalakshmi and Rajesh Kanna 2020)	India	Pf	Thin	Microscope + camera	Giemsa	50	NR	FoVs	No
D49 (Pathology Education International Resource 2014)	NR	Pf	Thin	NR	NR	NR	NR	Whole slide image	Yes
D50 (Sumi *et al.* 2021)	Indonesia	NR	Thick	Microscope + camera	Giemsa	NR	36	FoVs	No
D51 (Karimi *et al.* 2014)	Iran	Po, Pm	Thin	Microscope	Giemsa	27	27	FoVs	No
D52 (Wibisono *et al.* 2020)	Indonesia	NR	Thin	NR	NR	NR	100	FoVs	No
D53 (Hung *et al.* 2015)	NR	Pf, Pm, Po, Pv	NR	NR	NR	NR	96	FoVs	No
D54 (Adi *et al.* 2016)	NR	Pf	NR	Microscope + camera	NR	NR	152	FoVs	No
D55 (Rahmanti *et al.* 2016)	Indonesia	Pv	Thick	Microscope + camera	NR	NR	NR	Cell images	No
D56 (Xiong *et al.* 2015)	Singapore	NR	NR	Microscope + camera	Giemsa	4	>15 000	FoVs	No
D57 (Hendrawan *et al.* 2017)	Indonesia	Pf, Pv, Pm, Po	NR	NR	NR	NR	574	FoVs	No
D58 (Krappe *et al.* 2017)	NR	Pf, Pv, Pm	Thick	Microscope	NR	44	NR	Cell images	No
D59 (Sharma *et al.* 2022)	India	Pf, Pv	Thin	NR	Leishman	200	2800	FoVs	No
D60 (Nugroho *et al.* 2022)	Indonesia	Pf, Pv, Pm, Po	Thin	Microscope	NR	NR	559	FoVs	No
D61 (Delahunt *et al.* 2019)	South America, Africa, Asia, and London	Pf, Pv, Pm, Po	Thin	Microscope	Giemsa	765	323 000	FoVs	No
D62 (Kanakasabapathy *et al.* 2021)		Pf	Thin	Microscope	Giemsa	8	12 635, 11 397, 14 469	Cell images	Yes
D63 (Shaka 2021)	Tanzania	NR	Thick	Microscope + smartphone	Giemsa	100	10 000	FoVs	Yes
D64 (Zindi 2024)	Uganda	NR	NR	Microscope + smartphone	NR	NR	3925	FoVs	Yes
D65 (Muhammad *et al.* 2025)	Nigeria	NR	Thin	Microscope + smartphone	Giemsa, Field	100	24 712	FoVs	Yes
D66 (Adeleke *et al.* 2024)	Nigeria	Pm, Po, Pf	Thick and Thin	Microscope	Giemsa	NR	881	FoVs	Yes
D67 (Sukumarran *et al.* 2024)	Malaysia	Pf, Pv, Po, Pm, Pk	Thin	Microscope + camera	Giemsa	NR	472	FoVs	No
D68 (Li and Yang 2020)	NR	NR	NR	NR	NR	NR	843	Cell images	Yes
D69 (da Silva Ramos *et al.* 2024)	Brazil	Pv	Thin	Microscope	Giemsa	NR	27	FoVs	Yes
D70 (Chamidah *et al.* 2024)	Indonesia	NR	NR	NR	NR	NR	100	FoVs	No
D71 (Ozbilge *et al.* 2024)	Cyprus	Pf, Pv, Po	Thin	Microscope + smartphone	Giemsa	25	1081	FoVs	No

a Abbreviations: AOR, available on request; FoVs, field of views; NR, not reported; Pc, *Plasmodium cynomolgi*; Pf, *Plasmodium falciparum*; Pk, *Plasmodium knowlesi*; Pm, *Plasmodium malariae*; Po, *Plasmodium ovale*; Pv, *Plasmodium vivax*.

#### 4.3.1 Stain types

To identify malaria parasites, staining reagents were used to highlight cellular components in the selected studies. The staining reagents used ranged from Modified Romanowsky, Giemsa, May Grunwald-Giemsa, Leishman, and Field stains. The Giemsa stain accounts for 59% of all the datasets. Its stability and reliability make it the most widely utilized for identifying malarial parasites. Other reagents, such as Modified Romanowsky, May Grunwald-Giemsa, and Wright-Giemsa stains, were the least represented, as indicated in Fig. 6d. The Giemsa stain is widely used due to its stability, and it is the recommended and most reliable stain for identifying malaria parasites (WHO 2016c). Notably, 25% of the studies did not specify the staining reagent used.

**Figure 6.**
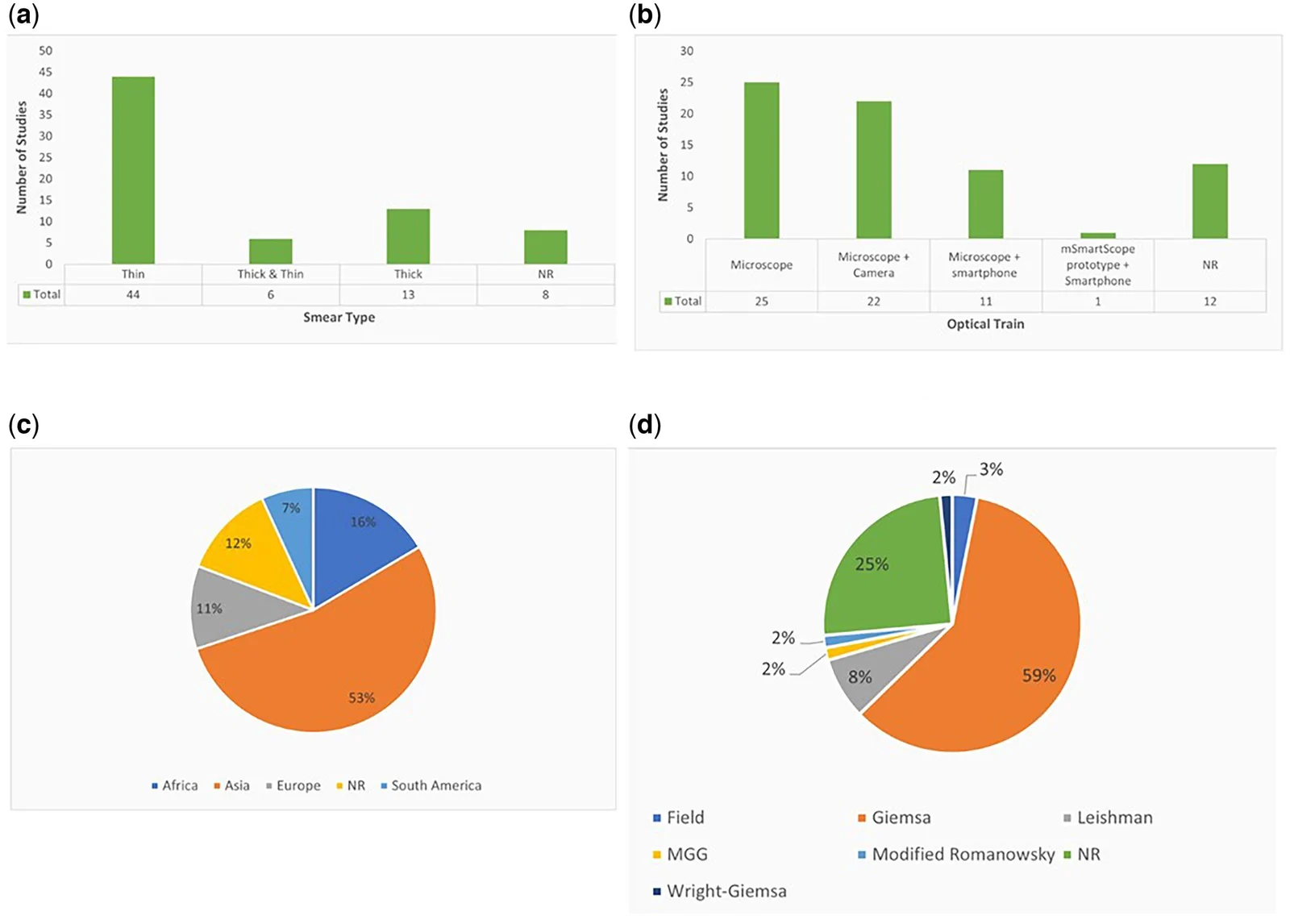
(a) Type of film used in selected studies, (b) imaging techniques used in selected studies, (c) distribution of datasets by continent, (d) staining techniques used in selected studies.

#### 4.3.2 Film types

The analysis revealed that six datasets featured both thick and thin blood film images. Thin blood films were more commonly used, with 44 studies relying exclusively on this type, compared to 13 studies that used only thick blood films. This suggests a research preference for thin blood film images in developing the automated malaria diagnostic systems. The distribution of film types used in the studies is illustrated in Fig. 6a.

#### 4.3.3 Imaging techniques

A range of equipment was used to capture the images for malaria diagnosis. Microscopes were the most common method, though some researchers attached smartphones to the microscope eyepiece to capture images. For example, Rosado *et al.* (2017) used a “mSmartScope” prototype paired with smartphones to capture their images. Other researchers attached cameras to microscopes for this task. For the image datasets featured in Davidson *et al.* (2021), six different microscopes were used by the research groups to obtain the images.

The statistics on the imaging techniques used are shown in Fig. 6b, with further details on the methods provided in Table 3. There is a clear need for more images to be generated to advance research in this field, as indicated by the image datasets discovered. While deep learning models for automated malaria diagnosis require large datasets to train neural networks (Karasu Benyes *et al.* 2022), it has been reported that the available datasets for malaria diagnosis are limited (Maqsood *et al.* 2021). Furthermore, Aqeel *et al.* (2023) noted the need for more comprehensive repositories of annotated image data and the importance of collecting and standardizing more data, given the various parasite species and the developmental stages.

**Table 3.** Imaging techniques used in the selected studies.^a^

Identifier	Optical train	Microscope type	Magnification	Image size
D1	Microscope + smartphone	Olympus C × 23 microscope	NR	NR
D2	Microscope + Camera	Brunel SP150 microscope	1000×	1024 × 768
D3	Microscope	A×io Zeiss Scope A1 Optical microscope	NR	2056 × 2452
D4	Microscope	ZEISS Axio Imager M2 microscope	100×	1388 × 1040
D5	Microscope	×SZ-107 series microscope	100×	NR
D6	Microscope + smartphone	Olympus microscope	100×	3264 × 2448
D7	Microscope	Brightfield microscope (Olympus B × 63)	100×	NR
D8	Microscope + Camera	Olympus B × 43	100×	2400 × 1800
D9	Microscope	Leica DM2000 optical laboratory microscope	100×	2592 × 1944
D10	Microscope + smartphone	NR	NR	NR
D11	Microscope + smartphone	Light microscope	100×	3024 × 4032
D12	Microscope + Camera	Olympus B × 41 microscope	100×	574 × 764
D13	Microscope	VS120, Olympus Corp	100×	2048 × 2048
D14	Microscope	Leica DLMA microscope	100×	1200 × 900
D15	Microscope	NR	100×	2048 × 1536
D16	Microscope	Light microscope	100×	1500 × 2100 × 3
D17	Microscope + Camera	Olympus C × 41 Microscope	100×	3136 × 2352
D18	Microscope + Camera	NR	100×	2592 × 1944
D19	Microscope + Camera	Light microscope	100×	640 × 480
D20	Microscope	NR	100×	1500 × 1000
D21	mSmartScope prototype + Smartphone	NR	NR	1944 x 2592 - 1840 x 3264 pixels
D22	Microscope	NR	100×	NR
D23	Microscope	NR	100×	352 x 288
D24	Microscope + Camera	Olympus BX51 microscope	NR	1360 x 1024
D25	Microscope + Camera	OMAX MD827S30 Binocular microscope	100×	NR
D26	Microscope + Camera	Leica DLMA microscope.	100×	800 x 600
D27	Microscope	Leica (DM750) microscope	100×	NR
D28	Microscope + Camera	Olympus BX43 microscope	100×	2400 × 1800
D29				
D30	Microscope + Camera	Axio Imager Z2, Carl Zeiss Microscopy AG, Jena, Germany	100×	1280 × 1024
D31	NR	NR	NR	NR
D32	Microscope + smartphone	NR	NR	NR
D33	Microscope + Camera	Leica ICC50 W microscope	NR	NR
D34	Microscope + smartphone	Olympus BX51 optical microscope	500×	NR
D35	Microscope	NR	100×	1280 × 960
D36	Microscope	XSZ-107 series microscope	100×	NR
D37	Microscope	NR	100×	NR
D38	Microscope, Microscope + Camera	NR	NR	NR
D39	Microscope	NR	400x	540 × 960
D40	Microscope + smartphone	Mobile Microscope Model	100×	NR
D41	Microscope + camera	Olympus CX31 microscope	NR	1920 × 1200
D42	NR		NR	2560 × 1920
D43	Microscope + camera	Olympus BX51 microscope	NR	1360 × 1024
D44	Microscope + camera	Olympus (BX51) Microscope	NR	NR
D45	NR	Optilab camera	100×	1600 × 1200
D46	Microscope + camera	Olympus BX51 microscope	100×	4080 × 3072
D47	NR	NR	NR	4032 × 3024
D48	Microscope + camera	Olympus CX21i bright field microscopic stage	100×	NR
D49	NR	NR		NR
D50	Microscope + camera	NR	NR	1600 × 1200
D51	Microscope	Olympus BX41 Microscope	100×	
D52	NR	NR	100×	1280 × 960
D53	NR	NR	100×	
D54	Microscope + camera	NR	100×	400 × 320
D55	Microscope + camera	NR	NR	NR
D56	Microscope + camera	Olympus BX51 microscope	100×	NR
D57	NR	NR	NR	256 × 256
D58	Microscope	NR	NR	NR
D59	NR	NR	100×	1500 × 1000
D60	Microscope	Optilab digital microscope camera	100×	960x1280
D61	Microscope	Motic EasyScan-Go	NR	2048 × 1536
D62	Microscope	Benchtop microscope, a portable stand-alone 3D-printed microscope and a smartphone-based microscope	NR	NR
D63	Microscope + smartphone	Olympus CX 21 microscope	100×	NR
D64	Microscope + smartphone	NR	NR	NR
D65	Microscope + smartphone	NR	NR	750 × 750
D66	Microscope	NR	100×	NR
D67	Microscope + camera	Olympus BX53 microscope	100×	NR
D68	NR	NR	400×	NR
D69	Microscope	NR	100×	1240 × 1645
D70	Microscope	NR	NR	600 × 512
D71	Microscope + smartphone	Light microscope	100×	640 × 640

a Abbreviation: NR, not reported.

This study also noted that some essential information about the image datasets was not provided by the researchers.

#### 4.3.4 Data availability

While some datasets discovered are publicly accessible, the availability of datasets from 42 studies remains unspecified. As previously mentioned, there is a clear need for publicly available and annotated image datasets to advance research in this domain. Table 4 outlines the data availability for each dataset, including the Digital Object Identifier (DOI) for each article. In cases where the DOI is unavailable, the article’s title is listed in its place.

**Table 4.** Dataset availability.^a^

Identifier	Data availability	Dataset link	Doi/title
D1	Publicly available	https://data.lhncbc.nlm.nih.gov/public/Malaria/MalariaScreener/index.html	https://doi.org/10.1186/s12936-023-04446-0
D2	Publicly available	https://air.ug/microscopy_dataset/	10.1201/9781315215723-10
D3	Publicly available	https://www.researchgate.net/publication/359064205_Image_features_for_quality_analysis_of_thick_blood_smears_employed_in_malaria_diagnosis	https://doi.org/10.1186/s12936-022-04064-2
D4	AOR		https://doi.org/10.1016/j.bspc.2022.103931
D5	AOR		https://doi.org/10.1007/s00521-021-06602-6
D6	Publicly available	https://drive.google.com/drive/folders/1p45Dt-BJy8hhoI-rYnhcaL6IMl5FsFL-	https://doi.org/10.3390/a14010017
D7	Publicly available	https://rdr.ucl.ac.uk/articles/dataset/Giemsa_Stained_Thick_Blood_Films_for_Clinical_Microscopy_Malaria_Diagnosis_with_Deep_Neural_Networks_Dataset_/12173568	10.1002/ajh.25827
D8	Publicly available	Uninfected: https://data.mendeley.com/datasets/c37wnbbd3c/1 Infected: https://data.mendeley.com/datasets/2v6h4j48cx/1	doi : 10.3390/e22060657
D9	Publicly available	https://github.com/andrealoddo/MP-IDB-The-Malaria-Parasite-Image-Database-for-Image-Processing-and-Analysis	10.1007/978-3-030-13835-6_7
D10	Publicly available	https://ceb.nlm.nih.gov/repositories/malaria-datasets/	10.7717/peerj.4568
D11	Publicly available	https://data.lhncbc.nlm.nih.gov/public/Malaria/NIH-NLM-ThickBloodSmearsPV/NIH-NLM-ThickBloodionsPV.zip Thin—TBD	https://doi.org/10.3390/diagnostics11111994
D12	AOR		10.32604/iasc.2022.024361
D13	Publicly available	https://ai.cdc.gov.tw/datasets/	10.1001/jamanetworkopen.2020.0206
D14	NR		10.1007/978–981-15–5281-6_57
D15	NR		https://doi.org/10.1016/j.patrec.2020.07.002
D16	NR		10.1109/AFRICON46755.2019.9133937
D17	NR		10.1109/WiSPNET.2017.8299974
D18	NR		10.1007/978–3-319-75420-8_45
D19	NR		10.1007/978-3-319–75193-1_23
D20	NR		10.1007/s00521-017-2937-4
D21	NR		10.3390/s17102167
D22	NR		Identification Of Malaria Disease and Its Stadium Based On Digital Image Processing
D23	NR		Unsupervised identification of malaria parasites using computer vision
D24	NR		10.1109/KST.2016.7440501
D25	NR		APDS (automated parasite detection system) for field malaria diagnosis in the Philippines
D26	NR		10.1063/1.4915828
D27	NR		10.1111/jmi.12206
D28	NR		10.1136/jclinpath-2019-206419
D29	NR		10.3389/fmed.2023.1117192
D30	Publicly available	http://fimm.webmicroscope.net/research/momic/mamic	10.1371/journal.pone.0104855
D31	NR		10.1109/icodse.2014.7062676
D32	Publicly available	https://doi.org/10.6084/m9.figshare.22679839	https://doi.org/10.1016/j.patter.2023.100806
D33	NR		https://doi.org/10.1016/j.compmedimag.2020.101845
D34	NR		https://doi.org/10.1016/j.imu.2022.101132
D35	NR		10.18517/ijaseit.9.4.4843
D36	NR		https://doi.org/10.1016/j.cmpb.2022.106996
D37	NR		10.3390/diagnostics11030527
D38	NR		10.1017/S2633903X21000015
D39	NR		10.1063/1.4976918
D40	NR		10.1109/OJEMB.2023.3328435
D41	Not publicly available to be shared		https://doi.org/10.1093/ofid/ofad469
D42	NR		https://doi.org/10.1007/978-981-13-3441-2_9
D43	NR		10.1109/KST.2018.8426105
D44	NR		10.4066/biomedicalresearch.29-18-970
D45	NR		http://doi.org/10.11591/ijeecs.v13.i2.pp721-728
D46	NR		10.1109/ACCESS.2020.2990497
D47	NR		https://doi.org/10.1145/3399637.3399641
D48	NR		https://doi.org/10.1007/s11042-019-7162-y
D49	Publicly available	http://peir-vm.path.uab.edu/wsi.php?slide=IPLab11Malaria	Pathology Education Informational Resource (PEIR) Digital Library
D50	NR		https://doi.org/10.1051/bioconf/20214104002
D51	NR		10.3109/00365548.2014.880186
D52	NR		https://doi.org/10.1145/3429789.3429825
D53	NR		10.1007/s40846-015–0101-0
D54	NR		Adi *et al.* 2016
D55	NR		10.1109/ICICI-BME.2015.7401339
D56	NR		10.1002/9781118715321.ch8
D57	NR		10.1109/SIET.2017.8304114
D58	NR		https://doi.org/10.1117/12.2249845
D59	NR		https://doi.org/10.1080/03772063.2020.1787238
D60	NR		10.48550/arXiv.2211.15105
D61	Not available		https://doi.org/10.48550/arXiv.1908.01901
D62	Publicly available	https://osf.io/3kc2d/	10.1038/s41551-021–00733-w
D63	Publicly available	https://zenodo.org/records/4314960	https://doi.org/10.5281/zenodo.4314960
D64	Publicly available	https://drive.google.com/file/d/16T40TdpaB8VXohm50SySREwrzbuPcJBC/view? usp=sharing	–
D65	Publicly available	https://zenodo.org/doi/10.5281/zenodo.13763938	https://doi.org/10.1016/j.bspc.2024.106869
D66	Publicly available	https://data.mendeley.com/datasets/6zpxnhjxzz/1	10.1016/j.dib.2024.110950
D67	AOR		10.1186/s13071-024–06215-7
D68	Publicly available	https://data.mendeley.com/datasets/38jtn4nzs6/2	10.17632/38jtn4nzs6.2
D69	Publicly available	https://github.com/JonathanRamos/PlasmodiumAI/tree/master/Datasets/Exams/FIOCRUZ	https://doi.org/10.1371/journal.pcbi.1012327
D70	NR		https://doi.org/10.28919/cmbn/8578
D71	NR		10.1109/ACCESS.2024.3393410

a Abbreviations: NR, not reported; AOR, available on request.

#### 4.3.5 Study location

The datasets identified originate from various regions, with 12 coming from Africa, while the remainder are from parts of Asia, South America, and Europe. Table 5 details the country, city, and study area as reported. As shown in Fig. 6b, a large proportion of the datasets originated from Asia, while 12% of the dataset origins were not reported. 16% came from Africa. This highlights the need for more datasets from malaria-endemic regions, particularly Africa. This observation is substantiated by Kassim *et al.* (2021), who emphasized the importance of evaluating automated malaria diagnostic models using data from different global regions to assess and ensure their adaptability to diverse datasets. Variability in medical images, including the devices used for capturing, the lack of standardization in medical practices, and patient genetic and phenotypic characteristics, along with parasite species and staining variations, influences the generalizability of machine learning models (Kuo *et al.* 2020, Maleki *et al.* 2023). The generalizability of these models is essential for real-world deployment and application in malaria diagnosis.

**Table 5.** Geographical information of the selected studies.^a^

Identifier	Country	City	Study area
D1	Sudan	Alsororab (SOR) area and Slanj (GS) area	Institute of Endemic Diseases, University of Khartoum, Sudan (IEND)
D2	Uganda	Kampala	Makerere AI Lab, Makerere University
D3	Columbia	NR	NR
D4	Brazil	NR	Instituto Nacional de Pesquisa da Amazˆonia– INPA
D5	Pakistan	Lahore	NR
D6	Uganda	Kampala	Mulago National referral hospital
D7	Nigeria	Ibadan	University College Hospital (UCH)
D8	Spain	Barcelona	Hospital Clínic of Barcelona
D9	Switzerland	Lausanne	Centre Hospitalier Universitaire Vaudois (CHUV)
D10	Bangladesh	Chittagong	Chittagong Medical College Hospital
D11	Bangladesh	Chittagong	Chittagong Medical College Hospital
D12	Malaysia	Kelantan	Hospital Universiti Sains Malaysia, Kubang Kerian
D13	Taiwan	NR	Taiwan CDC Biobank
D14	Malaysia	NR	Medical Microbiology and Parasitology Department, Hospital Universiti Sains Malaysia
D15	India	West Bengal	Midnapore Medical College and Hospital (MMCH), West Bengal
D16	Ghana	Accra	Noguchi Memorial Institute for Medical Research
D17	India	Surat	NR
D18	Philippines	Palawan	College of Public Health, University of the Philippines
D19	Spain	Barcelona	Microbiology department, Vall d’Hebron Hospital, Barcelona
D20	India	Silchar	Silchar Medical College and Hospital
D21	Portugal	NR	Instituto Nacional de Saúde Dr. Ricardo Jorge, Portugal
D22	Indonesia	NR	Central Health Laboratory North Sumatera Province
D23	NR	NR	NR
D24	Thailand	NR	NR
D25	Philippines	NR	Brgy. Luzviminda, San Rafael, and Puerto Princesa City Proper.
D26	Malaysia	NR	Department of Microbiology and Parasitology, Hospital Universiti Sains Malaysia (HUSM) and Hospital Universiti Kebangsaan Malaysia (HUKM).
D27	India	West Bengal	Midnapur Medical College and Hospital; and Medipath Laboratory, Midnapur, West Bengal, India
D28	NR	NR	NR
D29	China	Wuhan	Department of Schistosomiasis and Endemic Diseases, Wuhan City Center
D30	Finland	Helsinki	Helsinki University Central Hospital Laboratory
D31	Indonesia	NR	Eijkman Institute for Molecular Biology, Indonesia
D32	Sierra Leone	NR	Sierra Leone-China Friendship Hospital and the Rokupa Government Hospital
D33	NR	NR	Interstate blood bank (USA)
D34	France	NR	Parasitology lab of the Pitíe-Salpˆetri′ere Malaria NRC (National Reference Center) and the hematology lab of Saint Antoine Hospital
D35	Indonesia	NR	Eijkman Institute for Molecular Biology
D36	Pakistan	Punjab	Province of Punjab
D37	Korea	NR	Korea University Guro Hospital
D38	NR	NR	NR
D39	NR	NR	Hydas World Health
D40	Sudan	White Nile State	Alkawa Hospital, Alkawa, White Nile State, Sudan
D41	China	NR	Peking Union Medical College Hospital and Yunnan Institute of Parasite Diseases
D42	Indonesia	Jakarta	Bina Medical Support Services (BPPM)
D43	Thailand	NR	National Center for Genetic Engineering and Biotechnology (BIOTEC)
D44	India	Manipal	Haematology Lab, Kasturba Medical College (KMC), Manipal Academy of Higher Education (MAHE), Manipal
D45	Indonesia	NR	Department of Parasitology, Faculty of Medicine, Universitas Gadjah Mada
D46	Thailand	Pathum Thani	Protein-Ligand Engineering and Molecular Biology laboratory at the National Center for Genetic Engineering and Biotechnology (BIOTEC)
D47	China	Wuhan	Central South Hospital of Wuhan University
D48	India	Chennai	Tagore Medical College and Hospital, Chennai, India
D49	NR	NR	University of Alabama at Birmingham, Department of Pathology
D50	Indonesia	NR	Parasitology Laboratory, Faculty of Medicine, Universitas Gadjah Mada
D51	Iran	NR	NR
D52	Indonesia	NR	Eijkman Institute of Molecular Biolog
D53	NR	NR	China Medical University, China Medical University Hospital, and Taichung Veterans General Hospital in Taiwan
D54	NR	NR	NR
D55	Indonesia	NR	Dinas Kesehatan Provinsi Jawa Timur (East Java Health Department)
D56	Singapore	NR	NR
D57	Indonesia	NR	Balai Besar Laboratorium Kesehatan (Center for Health Laboratory) Indonesian Ministry of Health Surabaya
D58	NR	NR	Bernhard Nocht Institute for Tropical Medicine (BNITM)
D59	India	Assam	Pathological clinic of Cachar district, Assam, India
D60	Indonesia	NR	Eijkman Institute and the medical faculty of UGM
D61	South America, Africa, Asia, and London	NR	
D62	NR	NR	Massachusetts General Hospital (MGH) IVF laboratory and the MGH clinical pathology laboratory
D63	Tanzania	White Nile State	The University of Dodoma and Benjamin Mkapa Hospital Research Center
D64	Uganda	NR	NR
D65	Nigeria	Kano State	Asiya Bayero pediatric hospital, kano state, Nigeria and Murtala Muhammad specialist hospital
D66	Nigeria	Osun State, Oyo State and Cross River	Bowen University Hospital Iwo and Bowen University Teaching Hospital Ogbomoso and Cross river state
D67	Malaysia	NR	Malaria Research Centre, UNIMAS, Sarawak (MRC-UNIMAS)
D68	NR	NR	NR
D69	Brazil	Porto Velho, Rondônia	Fundação Oswaldo Cruz (FIOCRUZ)
D70	Indonesia	Surabaya	Department of Clinical Pathology, Faculty of Medicine, Airlangga University
D71	Cyprus	NR	Department of Parasitology, Manisa Celal Bayar University, and the Microbiology Laboratory of Near East University Hospital

a Abbreviation: NR, not reported.

The systematic review carried out showed limitations in data availability, metadata consistency, and geographic representation of datasets. To address this, the next section introduces an open-source interactive webpage developed to be a resource for discovering diverse blood film image datasets for malaria diagnosis.

## 5 Data accessibility and interactive mind map

In automated malaria diagnosis, data accessibility remains a challenge. From the datasets discovered in the study, several datasets were either not available due to the loss of datasets or accessible only upon request. These data and metadata accessibility issues will be a challenge for reproducibility and the benchmarking of robust machine learning models. The National Library of Medicine have made most of their generated annotated image datasets accessible, and this includes the thick blood film *P. falciparum*-infected images from 150 patients, the thin blood film uninfected and *P.* falciparum-infected images from 193 patients, among others. Other image datasets can be found on Kaggle, Mendeley data, Zenodo, and the research group’s webpage. With all these efforts by the researchers, there is still a need for many image datasets to be made publicly available (Maturana *et al.* 2022).

In biomedical image analysis, data accessibility is very important. In the scientific community, the FAIR principles (Findability, Accessibility, Interoperability, and Reusability) have been emphasized for data sharing and standardization (Wilkinson 2016). To follow these principles, some of the available datasets that have the license for redistribution have been hosted on Zenodo for easy access, and the link can be accessed at https://zenodo.org/records/16958076. This study also introduces an interactive mind map to facilitate datasets to be easily discovered by researchers in this domain.

The findings of this research are being presented interactively through a user-friendly mind map developed with the static Jekyll Cayman theme on GitHub pages. A dedicated repository “Malaria_Blood_Film_Images” was created to store markdown files detailing individual blood film datasets. The Jekyll Cayman theme was integrated into the repository, complete with its configuration, layout, and style files for webpage customization. An interactive HTML landing page was also designed and deployed as a GitHub page. It categorizes datasets by film type, *Plasmodium* species, staining method, imaging technology, geographical details and availability. It enables users to visually explore datasets and directly access their source URLs or DOIs. This approach provides a user-friendly alternative to traditional static tables, reducing search time and encouraging dataset reuse.

The structure of this repository allows for updates from the scientific community. Each dataset’s metadata information has been documented in markdown files, allowing for easy revision and contributions by other users. The choice of GitHub for this is to ensure that the repository remains open source and can also benefit from version control (Russell *et al.* 2018). Just like was demonstrated by the MalariaScreener datasheet (National Library of Medicine 2020), Centralizing these datasets’ information will enhance their use for training AI models.

As the field increasingly moves towards generalizable, cross-site diagnostic tools, access to a broad and well-documented pool of training data becomes essential. The mind map contributes to this goal by lowering the barrier for researchers, especially those in low-resource settings, to discover and leverage valuable image datasets for automated diagnosis development. The repository is publicly available and maintained at https://github.com/itunuisewon/Malaria_Blood_Film_Images, with the live interactive site accessible at https://itunuisewon.github.io/Malaria_Blood_Film_Images/.

A detailed guide for navigating and using the interactive mindmap webpage has been provided in Supplementary File 1, available as supplementary data at Bioinformatics Advances online. This includes a brief explanation of the nodes and how to interact with them.

## 6 Challenges and future direction

In the automated diagnosis of malaria, machine learning has seen significant progress, but there is a major need to translate all this progress into solutions that can be deployed in clinical settings.

From our study, one of the major limitations seen is the inconsistency in the formats of image datasets. Datasets vary in formats, magnification and even annotation types. This inconsistency is a major hindrance to the integration of images across studies, thereby obstructing reproducibility (Straiton 2024). This lack of standardization is a result of differences in data collection, annotation and regional practices. Another challenge seen is the absence of some information, like imaging equipment, film type, and staining method, in some of the datasets. Having accurate metadata is important in understanding the data and training robust models. In addition, many of the datasets only provide binary labels, with just a few offering species differentiation or lifecycle-stage annotations.

The geographic bias is another major challenge. Many of the publicly available image datasets originate from a limited number of countries, mainly in Asia. Despite African countries bearing a high malaria burden, they remain underrepresented. This bias can reduce the effectiveness of models when they are deployed in underrepresented populations, which will, in turn, cause diagnostic errors (Mehrabi *et al.* 2022, Kabore and Guel 2024). The varying prevalence of species, including coinfection, is often not shown in training datasets, and this can lead to limited adaptability to clinical contexts.

To address these challenges in developing robust and generalizable models, we propose establishing a repository specific to malaria image datasets to host standardized datasets with accurate and complete metadata, similar to The Cancer Imaging Archive (TCIA). A standardized metadata schema can also be adopted to ensure that all imaging information is included in the datasets. Efforts can also be made to develop tools that can correct for the colour and contrast variations that can be caused using different stains and data heterogeneity.

## 7 Conclusion

In conclusion, this study provided an overview of the conventional microscopy method for malaria diagnosis, focusing on the curation of blood film image datasets, as data plays a vital role in developing automated diagnostic systems (Akinrinmade *et al.* 2023). The study revealed several limitations in the curated datasets. These include inconsistencies in image dataset formats, variations in magnification and annotation types, a notable absence of essential metadata (such as imaging equipment and staining methods) in many datasets, and a significant geographic bias. Furthermore, data accessibility remains a challenge, as many datasets are either unavailable or accessible only upon request, hindering reproducibility and the benchmarking of robust models. Larger, well-annotated image datasets are required to improve the classification accuracy of machine learning models for automated malaria diagnosis. To address these critical limitations and support the advancement of automated malaria diagnosis, this work introduces an open-source, interactive website (mind map). This centralized resource is designed to enhance data accessibility and discoverability by categorizing datasets by film type, *Plasmodium* species, staining method, imaging technology, geographical details, and availability, allowing researchers to easily explore and access source URLs or DOIs. It will act as a valuable resource for researchers and developers working on malaria detection and diagnosis. The interactive mind map is publicly accessible at https://itunuisewon.github.io/Malaria_Blood_Film_Images/, and the corresponding Zenodo repository containing the curated datasets is available at https://zenodo.org/records/16958076.

## Supplementary Material

vbaf276_Supplementary_Data

## Data Availability

The data underlying this article are available on GitHub at https://github.com/itunuisewon/Malaria_Blood_Film_Images.
