# Supplementary material for: An interactive mindmap of blood film images for automated malaria diagnosis: a comprehensive metadata repository of available datasets
Source: Bioinform Adv. 2025 Nov 4;6(1):vbaf276. doi: 10.1093/bioadv/vbaf276 (PMC13660762; doi:10.1093/bioadv/vbaf276)
Supplement: Supplementary file 1 [file vbaf276_supplementary_data.docx]

***Supplementary Materials***

An Interactive Mindmap of Blood Film Images for Automated Malaria Diagnosis: A Comprehensive Review of Available Datasets

Itunuoluwa Isewon^1,2,3,*^, Faith Adegoke^1,2,3^, Charles B. Delahunt^4^, and Jelili Oyelade^1,2,3^

^1^Department of Computer and Information Sciences, Covenant University, P.M.B. 1023, Ota, Ogun State, Nigeria

^2^Covenant Applied Informatics and Communication African Centre of Excellence (CApIC-ACE), Covenant University, P.M.B. 1023, Ota, Ogun State, Nigeria

^3^Covenant University Bioinformatics Research (CUBRe), Covenant University, P.M.B. 1023, Ota, Ogun State, Nigeria

^4^Global Health Labs, Bellevue, WA, United States

*Corresponding author. Department of Computer and Information Sciences, Covenant University, P.M.B. 1023, Ota, Ogun State, Nigeria. E-mail: itunu.isewon@covenantuniversity.edu.ng

**Navigation Guide for the Interactive Mind Map for Malaria Blood Smear Images**

The interactive mind map was designed to help researchers locate and explore blood film image datasets. This repository categorizes the datasets using relevant metadata for automated diagnosis. This guide was made to show how the interactive tool can be navigated.

**Figure S1: The interactive mindmap**

**The** **Main Node: Blood Smear Images**

This is the **central hub** of the mind map. From here, five main branches extend, each representing a different dataset metadata and they are:

1. **Access**
2. **Smear Type**
3. **Stain Type**
4. **Species**
5. **Demography**

**The Branches**

1. **Access:** This branch categorizes datasets based on how they can be obtained:

- **Open Access**: These datasets are freely available to the public without any restrictions. Clicking on this node will lead you to datasets that have their markdown document, which contains details about them.
- **Controlled Access**: These include datasets that may require permission to access.

2. **Smear Type:** This branch helps you filter datasets by the type of blood smear/film used:

- **Thin Blood Smear**: These datasets only contain thin blood smear/film images. It is ideal for species identification and parasite morphology.
- **Thick Blood Smear**: These are datasets that contain thick blood smear/film images. It is used for parasite detection and parasitemia estimation.
- **Thick & Thin Blood Smear**: These datasets contain both types of smear/film.

3. **Stain Type**: This branch classifies datasets by the staining technique used to enhance parasite visibility:

- **Giemsa Stain**: The most widely used and WHO-recommended stain.
- **Field Stain**: A rapid stain commonly used in field settings.
- **Leishman Stain**: Another Romanowsky-type stain, popular in some regions.
- **May-Grünwald Giemsa Stain**: A combination stain for enhanced nuclear contrast.
- **Wright-Giemsa Stain**: Used more in clinical haematology but also for parasites.
- **Modified Romanowsky Stain**: Variants adapted for specific research conditions.

4. **Species:** This branch identifies which *Plasmodium* species are represented in the datasets, and they are:

- ***Plasmodium falciparum***
- ***Plasmodium vivax***
- ***Plasmodium ovale***
- **Combined Species**: Datasets that contain mixed or multi-species infections, useful for species classification tasks.

5. **Demography**: This branch categorizes datasets based on their geographic origin.

- **Africa**
- **Asia**
- **Europe**
- **South America**

**How to Interact with the Mind Map**

- **Click on any node** to expand its child nodes.
- **Click on the category node** to view the list of datasets with links to their metadata such as smear type, stain, species, and access level.
- **Click on dataset names or links** to open their hosting platform (e.g., GitHub, publications, or data portals).

**Update and Contribution**

- The repository is updateable.
- Users can suggest new datasets or corrections via pull requests or GitHub Issues.
